# Purple corn extract alleviates 2,4-dinitrochlorobenzene-induced atopic dermatitis-like phenotypes in BALB/c mice

**DOI:** 10.1080/19768354.2021.1974938

**Published:** 2021-09-08

**Authors:** Huiwon No, Sang Hyun Nam, Hee Won Seo, JeongHyeon Seo, Soo-Hyun Park, Set-Byeol Kim, Jun-Sub Jung, Jongyeol Park, Jaekeun Choi, Jae-Yong Lee, Hong-Won Suh, Soon Sung Lim, Jin-Kyung Kim

**Affiliations:** a Department of Biomedical Science, Daegu Catholic University, Gyeongsan-Si, Republic of Korea; b Front Bio Inc., Chuncheon, Republic of Korea; c Maize Research Institute, Hongcheon-gun, Republic of Korea; d Department of Biochemistry, College of Medicine, Hallym University, Chuncheon, Republic of Korea; e Department of Pharmacology, College of Medicine, Hallym University, Chuncheon, Republic of Korea; f Department of Food Science and Nutrition, Hallym University, Chuncheon, Republic of Korea

**Keywords:** Atopic dermatitis, 2,4-dinitrochlorobenzene, purple corn, IgE

## Abstract

*Zea mays L.* (Poaceae), also known as purple corn, is an annual herbaceous plant that is grown as food for human consumption in a variety of forms, including cooking oils and sweeteners in processed food and beverage products. The purpose of this study was to determine whether a novel purple corn extract, FB801, might have an anti-atopic dermatitis (AD) effect on AD-like skin lesions induced by 2,4-dinitrochlorobenzene (DNCB) in BALB/c mice. Topical sensitization (1%) and challenge (0.3%) by DNCB were performed on the dorsal skin and right ear of BALB/c mice to induce AD. Following FB801 and dexamethasone administered orally, the severity of skin lesions was examined macroscopically and histologically. Serum levels of immunoglobulin E (IgE) and various cytokines were determined by enzyme-linked immunosorbent assay. Oral administration of FB801 significantly reduced typical symptoms of AD (erythema/bleeding, swelling, molting/erosion and scaling/drying), scratching frequencies, and the recruitment of inflammatory and mast cells. In addition, FB801 suppressed serum levels of IgE and T helper (Th)2 type cytokines such as interleukin (IL)-4 and IL-10 in DNCB-treated BALB/c mice. Furthermore, FB801 reduced the degradation of inhibitor of nuclear factor-κB proteins (NF-κB) in tumor necrosis factor (TNF)-α-stimulated human keratinocyte (HaCaT) cells. These results suggest that FB801 inhibited the development of the AD-like skin symptoms by regulating Th1 and Th2 responses in the skin lesions in mice and suppressing TNF-α induced NF-κB activation in HaCaT cells, suggesting that FB801 has potential application as an effective alternative therapy for the prevention and management of AD.

## Introduction

Atopic dermatitis (AD) is a chronic inflammatory skin disease that manifests as a skin hypersensitivity reaction that progresses by the activation of inflammatory cells associated with various allergic immune responses (Weidinger and Novak 2016; Kim et al. 2019). The prevalence of AD in children and adolescents has augmented over the past decade (Laughter et al. 2021). Mechanical annihilation of the skin barrier can be repaired by self-renewal of basal epithelial cells in the stratum corneum with infiltration of inflammatory cells related to wound healing such as neutrophils, macrophages, and fibroblasts. However, skin lesions repeatedly exposed to allergens can activate allergic responses by attracting specialized cell clones such as mast cells, eosinophils, dendritic cells, and T cells (Weidinger and Novak 2016; Kim et al. 2019). Patients with AD have immediate immunoglobulin E (IgE)-mediated hypersensitivity reactions (Custovic et al. 2015; Furue et al. 2017). Intense pruritus or itching is the most common and burdensome dermatologic feature of AD that can negatively impact the health-related quality of life of patients suffering from AD (Custovic et al. 2015; Weidinger and Novak 2016; Furue et al. 2017; Kim et al. 2019). It has been shown that aeroallergen exposure can lead to deteriorating AD symptoms (Gray et al. 2017). Overexpression of T helper (Th)2 cytokines has been observed in acute and subacute lesions of AD (Vangipuram and Tyring 2017). The clinical impact of key Th2 cytokines interleukin (IL)-4 and IL-13 on atopic dermatitis has been recently shown in clinical studies with dupilumab, a monoclonal antibody that blocks the IL-4/IL-13 receptor (Vangipuram and Tyring 2017; Rodrigues et al. 2019).

Various therapies are being developed to reduce the severity of AD pathologies, prevent further infections, and control the disease in the long term. Pharmacological approaches include the use of corticosteroids to reduce inflammation, antibiotics to eradicate infections induced by bacteria or other parasites, antihistamines to reduce pruritic symptoms, and calcineurin inhibitors to prevent eczema dissemination and diminish inflammation (Mayba and Gooderham 2017; Kusari et al. 2019). Phototherapy and immunomodulation by systemic administration of immunosuppressant drugs have also been proposed (Mayba and Gooderham 2017; Kusari et al. 2019). Despite the extensive use of these therapies, there is a demand for new treatment that can specifically target AD. As an alternative option, creating new effective drugs from natural products containing phytochemicals has been accepted. As a typical example, specific oat extracts can be used to alleviate AD in infants, thus reducing the use of topical corticosteroids (Grimalt et al. 2007). Anti-inflammatory agents containing *Vitis vinifera* extracts have been demonstrated to be effective in the treatment of AD (Abramovits and Perlmutter 2006).

Purple corn (*Zea mays* L.), also known as purple maize, is an annual grass in the family of Poaceae (grass family) originated from Peru but widely distributed throughout the world, mainly in markets of Asia, the United States, and Europe. Purple corn has a prodigious potential in food and pharmaceutical industries because of its high contents of bioactive compounds that are beneficial for health (Cristianini and Guillén Sánchez 2020). Several studies including our previous studies have shown that purple corn extract possesses anti-inflammatory, anti-adipogenic, and anti-diabetic effects (Kang et al. 2012; Kang et al. 2013; Intuyod et al. 2014; Huang et al. 2015). The purple corn (Saekso 4) used in our study is a new variety made through plant breeding at the Maize Research Institute (Gangwon-do, South Korea). The objective of the present study was to investigate the therapeutic effect of FB801, a novel purple corn extract, on AD-like skin lesions induced by 1-chloro-2,4-dinitro benzene (DNCB) in BALB/c mice and tumor necrosis factor (TNF)-α stimulated human keratinocytes.

## Materials and methods

### Preparation of purple corn extract, FB801

Dried purple corn [*Zea mays* L., (Saekso 4)] cobs were obtained from Maize Research Institute (Gangwon-do, South Korea). All solvents and other chemicals were purchased from Fisher Scientific (Fair Lawn, NJ, USA). Before extraction, dried purple corn cobs were chopped into small size (1–5 cm) with a chopping machine (NIRAV, Balaji Chowk, India) to facilitate extraction. Chopped purple corn cobs (approximately 140 g) were extracted with 30% ethanol (1.4 L) for 8 h at 50°C in a shaker (SJ-898SR-2) twice. The crude extract was filtered through a Whatman No.1 filter (6–10 μm) using a vacuum funnel. Solvent (ethanol) was evaporated to dryness at 45°C with a rotary evaporator (40–100 mbar). The extract was then freeze-dried at 50°C. The final extract with a serial number of FB801 (19.41 g) was obtained, having a yield of 13.86%.

### HaCaT cells

HaCaT cells, the immortalized human keratinocyte cell line, was kindly provided by Professor C. Kang (College of Veterinary Medicine, Gyeongsang National University) and were maintained in Dulbecco’s modified Eagle’s medium (DMEM) supplemented with 10% fetal bovine serum (FBS), penicillin (100 U/mL) and streptomycin(100 μg/mL) were obtained from Hyclone (Logan, UT, USA) at 37°C exposed to 5% CO_2_ in a humidified incubator.

### Measurement of cell viability

The effect of FB801 on HaCaT cell viability was evaluated by Cell Counting Kit (CCK)-8 (Dojindo Laboratories, Kumamoto, Japan). 1×10^4^ cells in 100 μL were seeded in each well of a 96-well plate and incubated for 12 h at 37°C with 5% CO_2_. Various concentrations of FB801 were dissolved in fresh medium and added to each well. After 24 h of incubation, 10 μL of CCK-8 reagent was added to each well, and the cells were further incubated for 2 h. The absorbance of each well was measured using a microplate reader at 450 nm (Tecan Sunrise^TM^, Tecan Group Ltd., Männedorf, Switzerland).

### Western blot analysis

HaCaT cells (1 × 10^6^ cells) were grown in culture medium on plates (100 mm diameter) for 12 h. They were then pretreated with indicated concentration of FB801 in a complete medium for 4 h and stimulated with recombinant human TNF-α (PeproTech Korea, Seoul, Korea) for 15 min. The protein concentration was determined using the bicinchoninic acid protein assay kit (Thermo Fisher Scientific, Waltham, MA, USA), and equal amounts of protein in the whole cell lysates were separated using sodium dodecyl sulfate (SDS)-polyacrylamide gel electrophoresis. Then, total protein was transferred to the polyvinylidene fluoride (PVDF) membrane (Bio-Rad Laboratories, Hercules, CA, USA) prior to blocking using 5% skim milk in Tris-buffered saline-Tween 20 (TBS-T) solution. The primary antibodies for extracellular signal-regulated kinase (ERK1/2, Cell Signaling Technology Inc., Beverly, MA, USA), phospho-ERK1/2 (Cell Signaling Technology Inc.), c-Jun N-terminal kinase (JNK1/2, Cell Signaling Technology Inc.), phospho-JNK1/2 (Cell Signaling Technology Inc.) p38 (Cell Signaling Technology Inc.), phospho-p38 (Cell Signaling Technology Inc.), inhibitor of nuclear factor-κB (NF-κB) proteins (IκB-α, Cell Signaling Technology Inc.), and β-actin (Sigma-Aldrich, St. Louis, MO, USA) were incubated with the PVDF membrane overnight at 4°C. Then, the secondary antibody (1:2000 in 5% BSA in TBS-T solution) was incubated for 1 h at room temperature (Cell Signaling Technology Inc.). All primary antibodies were diluted 1:1000 with 5% skim milk in TBS-T solution. The protein bands were then visualized using enhanced chemiluminescence reagent (Thermo Fisher Scientific) and DAVINCH-Chemi CAS-400SM (Davinch-k, Seoul, Korea). Band densities were measured using Total Lab software (Davinch-k).

### Experimental animals

Six-week-old female BALB/c mice purchased from Hyochang Science (Daegu, Korea) were housed in the Animal Resource Facility, Daegu Catholic University in accordance with the university’s Institutional Animal Care and Use Committee guidelines. All mice were maintained under standard conditions (alternating 12-h dark and 12-h light cycle, temperature of 24 ± 2°C, and relative humidity of 50 ± 10%). The animal protocol used in this study was approved by the Institutional Animal Care and Use Committee (approval number: CUC-2016-052).

### Induction of AD–like skin lesions using DNCB

After one week of acclimation, mice were divided into four groups (*n* = 5 per group): (1) Normal control (NC) group of mice were applied with acetone: olive oil mixture (3:1 vol/vol) and treated with normal saline; (2) DNCB (Sigma-Aldrich) group of mice were sensitized with DNCB and treated with normal saline; (3) DNCB + Dex group of mice were sensitized with DNCB with oral application of dexamethasone (Sigma-Aldrich) every three days (5 mg/kg body weights) as a positive control group; and (4) DNCB + FB801 group of mice were sensitized with DNCB and treated daily with FB801 at 30 mg/kg body weight. FB801 doses were selected and modified according to previous studies (data not shown).

**Figure 1.**
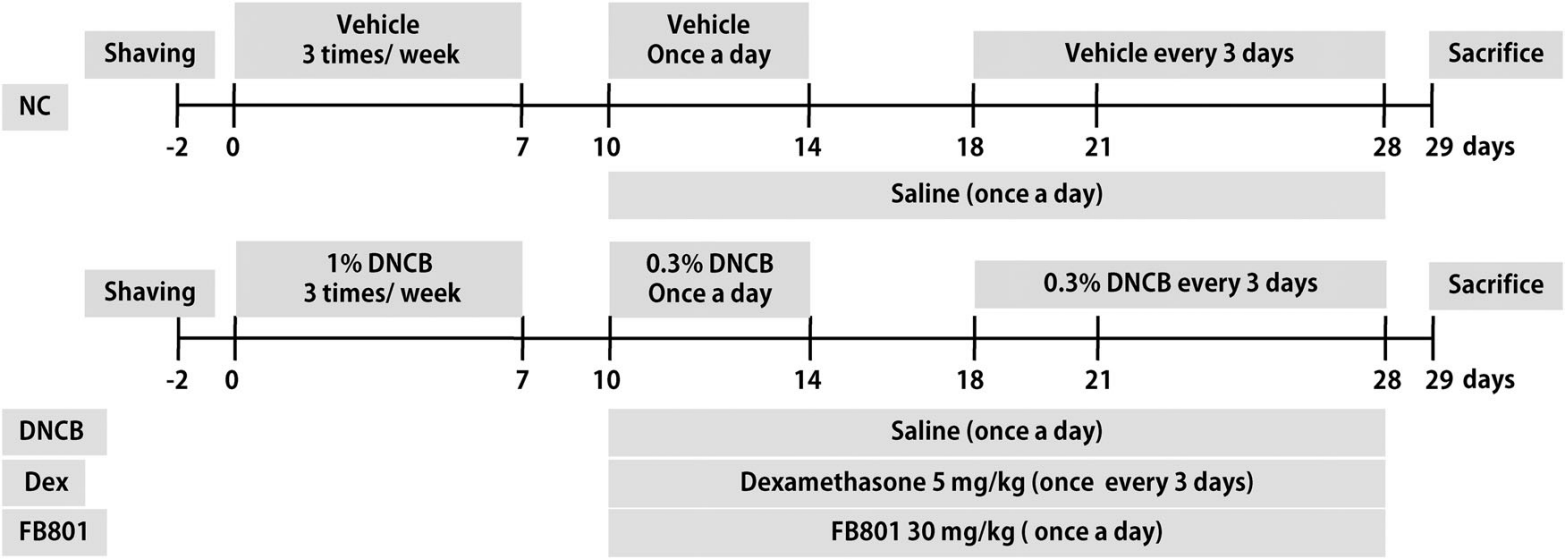
Experimental scheme showing the 2,4-Dinitrochlorobenzene (DNCB)-induced atopic dermatitis (AD) model. On days 0, 4, and 7, mice were sensitized with 200 and 20 μL of 1% DNCB or vehicle at their shaved back and right ear, respectively. Three days after the first sensitization, 0.3% DNCB was applied to challenge the dorsal skin (200 μL) and the right ear (10 μL) for 4 days. From day 18 onwards, 0.3% of DNCB or vehicle was applied to challenge the dorsal skin and right ear every three days until the end of experiments. AD-induced mice were co-treated with either FB801 once daily or dexamethasone every three days from day 10 onwards until the end of experiments.

AD–like skin lesions were induced in BALB/c mice according to a published method (Meng et al. 2019) with minor modifications. Briefly, an electric razor was used to completely remove dorsal hair of mice (an area of ∼4 cm^2^). Two days after hair removal, 200 µL 1% DNCB dissolved in a mixture containing acetone and olive oil (3:1 v/v) was dropped onto the dorsal skin and 20 µL 1% DNCB solution was dropped in right ears on days 0, 4, and 7. Three days after the first sensitization, 0.3% DNCB was applied to challenge the dorsal skin (200 μL) and the right ear (10 μL) for 4 days. Four days after the challenge period, 0.3% DNCB was applied once every three days to maintain AD–like symptoms (days 18–28). For the vehicle group, the same dose of the mixture comprising acetone and olive oil (3:1 v/v) was applied to the dorsal skin and ears of mice. One hour after each DNCB application, dexamethasone or FB801 was orally administered (days 10–28). The experimental schedule is summarized in Figure 1.

### Dynamic evaluation of dermatitis

Dermatitis in each mouse was observed and scored once a week according to the criteria described previously (Koszorú et al. 2019). Lesion severity scoring was used to estimate the severity of the dermatitis on back regions of mice each week, including scoring of erythema/hemorrhage, edema, excoriation/erosion, and scaling/dryness. The score of each clinical symptom ranged from 0 to 3 (none, 0; mild, 1; moderate, 2; and severe, 3). Total dermatitis score (maximum score 12) was the sum of individual scores.

Ear thickness was measured using a digital micrometer (Mitutoyo Corporation, Tokyo, Japan). The micrometer was applied near the tip of the ear just distal to the cartilaginous ridges, and the thickness was recorded in micrometers.

### Evaluation of scratching behavior

Each mouse was observed for 10 min and the number of scratches was recorded once a week. A scratching event was defined as rubbing dorsal skin and ears with hind paws of mouse. When continuous scratch time exceeded 3 s, it was recorded as two scratches. Mice with high and low number of scratches were excluded from the experiment. All measurements were performed by a single investigator to avoid inter-observer variation.

### Histopathological alterations

Dorsal skin samples were fixed in 10% buffered formalin for 24 h at room temperature, embedded in paraffin, and sectioned into 6-μm thick slices. Skin sections were stained with hematoxylin and eosin (H&E) according to the manufacturer's protocol. General tissue features were observed under a light microscope (Leica Biosystems Richmond Inc., Richmond, IL, USA). Toluidine blue staining was performed for measuring mast cell infiltration. The number of mast cells was counted in five randomly selected fields of view under a light microscope.

### Measurement of serum IgE and cytokines using ELISA

To measure serum IgE and cytokines, blood samples were centrifuged at 10,000 rpm for 15 min at 4°C. Serum was collected and stored at −80°C for further analysis. The levels of IL-4, interferon (IFN)-γ, IL-10 and IgE in serum were quantified using ELISA kits (eBioscience, San Diego, CA, USA.) according to the manufacturer’s protocols. Briefly, sample and standard solutions were transferred to 96-well plates pre-coated with the appropriate monoclonal antibodies, and then incubated at room temperature for 2 h. After thorough washing, horseradish peroxidase-conjugated secondary antibodies were added to each well, then incubated at room temperature for 1 h. After removal of the secondary antibodies, the substrate solution was added, and samples were incubated for another 30 min in the dark. The reaction was terminated by addition of stop solution, and absorbance was measured at 450 nm using a microplate reader (Tecan Sunrise^TM^).

The levels of IL-6 and TNF-α secreted by HaCaT cells were also measured using ELISA kits (eBioscience). The HaCaT cells were seeded into a 24-well plate at a density of 2×10^4^ cells per well, incubated overnight, and then pretreated with indicated concentration of FB801 for 4 h followed by stimulation with TNF-α (10 ng/mL) for 24 h. Cell culture supernatants were collected and the release of IL-6 and TNF-α was quantified using an ELISA kit according to the manufacturer's instructions.

### Statistical analysis

All statistical analyses were performed using Prism v.6.0 software (GraphPad Inc., La Jolla, CA, USA) and data were presented as mean ± SEM. Analyses of differences were performed using unpaired t-test or one-way ANOVA. Statistical significance was considered at *p* < 0.05, *p *< 0.01, and *p* < 0.001.

## Results

### Effects of FB801 on AD symptoms in DNCB-induced BALB/c mouse

**Figure 2.**
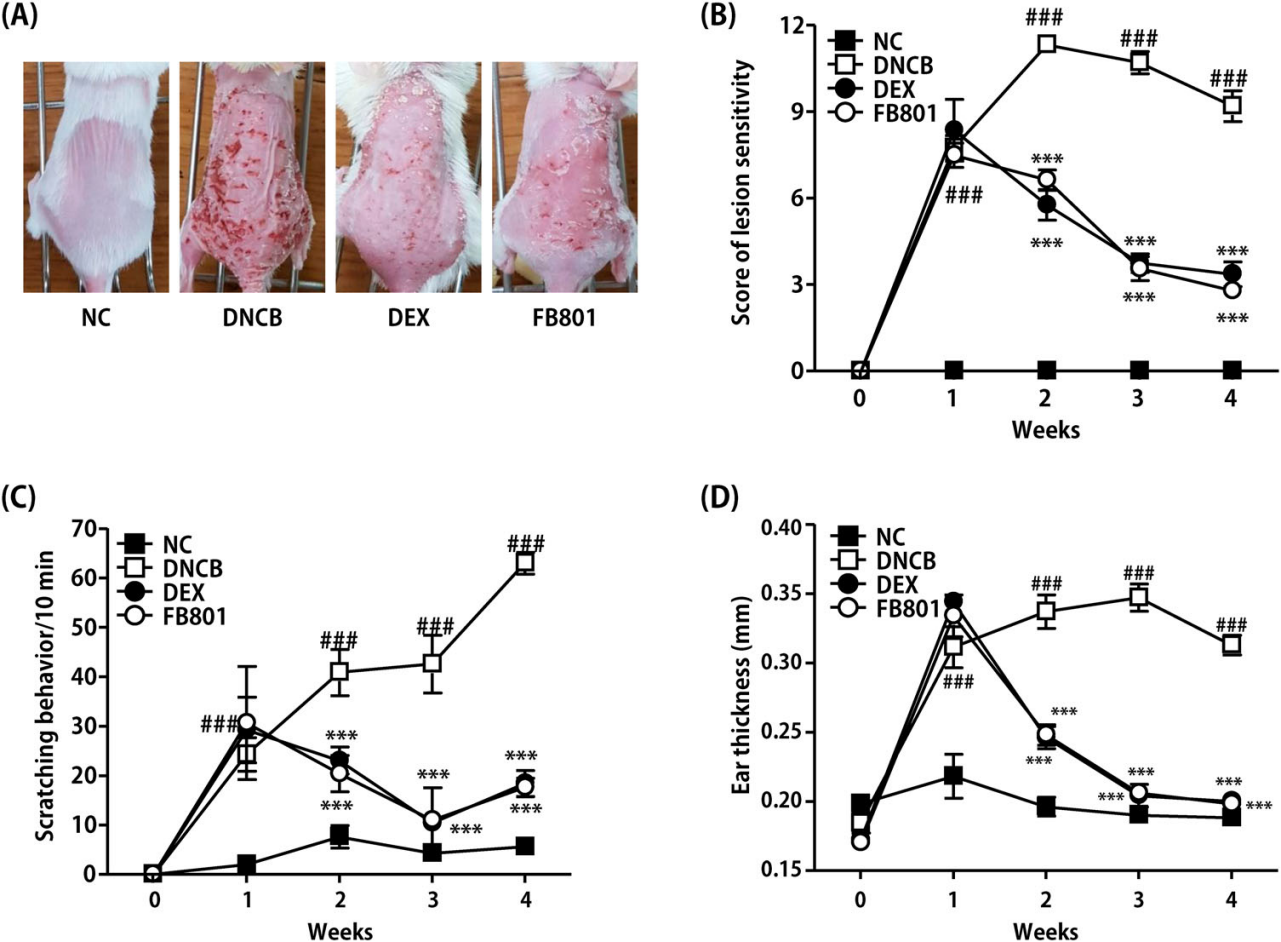
Effects of FB801 on atopic dermatitis (AD)-like clinical signs in 2,4-Dinitrochlorobenzene (DNCB)-treated mice. (A) To examine the severity of AD-like lesions, photographs of mouse dorsal skin were taken before mice were sacrificed on the 28th day of the experiment. (B) Skin severity scores of AD-like skin lesions in BALB/c mice. The total score is the sum of individual scores determined based on symptoms of erythema/hemorrhage, edema, scaling/dryness, and excoriation/erosion. (C) Scratching incidence and (D) Ear thickness were evaluated once a week. Data are expressed as mean ± SEM of five mice per group. ^###^*P *< 0.001 compared with the normal control (NC) group; ***P < 0.001 compared with DNCB-stimulated group.

We first examined whether FB801 might have beneficial effects on the pathogenesis of AD using DNCB-induced AD mice. The skin of mice in the DNCB group exhibited AD-like signs and symptoms that began with infiltrative erythema, edema, pruritus and hemorrhage, followed by erosion, scratching, excoriation, and dryness (Figure 2(A)), similar to clinical manifestation of AD. No alteration was observed for the back skin of normal control mice (Figure 2(A)). The severity of dorsal skin lesions evaluated with reference to known standards was significantly reduced in the FB801-treated group (Figure 2(B)). Scratching frequency was observed to monitor the itching behavior of each mouse. FB801 and dexamethasone treatments significantly inhibited DNCB-induced itching sensation in experimental mice (Figure 2(C)). When ear thickness was measured over time, we found that the thickness increased as atopic dermatitis developed as shown in Figure 2(D). Consistent with the severity of dorsal skin lesions and itching behavior, ear thickness was significantly reduced after oral administration of FB801.

**Figure 3.**
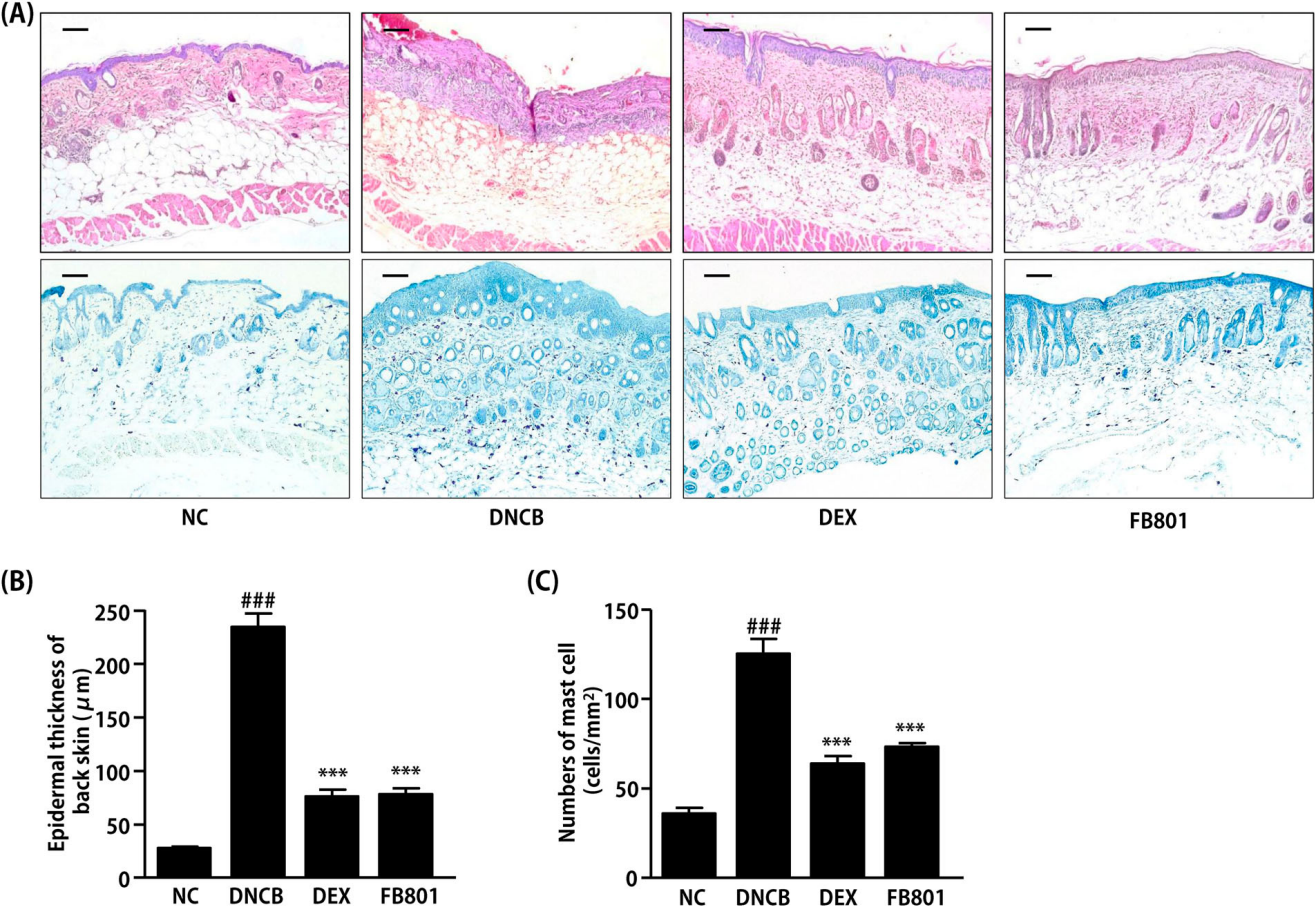
FB801 reduces dermal infiltration of inflammatory cells and mast cells in 2,4-Dinitrochlorobenzene (DNCB)-treated BALB/c mice. Cut dorsal skin was fixed with 10% buffered formalin, cut into 6-μm sections, and stained with hematoxylin and eosin (H&E, upper panel, bar: 100 µm) or toluidine blue (lower panel, bar: 100 µm). Photographs of each group represent images of all mice (*n* = 5). Images were taken at 200× magnification. (B) Epidermal thickness was analyzed for H&E stained sections. (C) The number of infiltrated mast cells in the dermis was examined after toluidine blue staining of skin sections. It was counted in five fields per mice. Data are expressed as mean ± SEM of five mice per group. ^###^*P *< 0.001 compared with the normal control (NC) group; ***P < 0.001 compared with DNCB-stimulated group.

Since DNCB treatment caused hyperkeratosis and hypertrophy of the dorsal skin tissue, thicknesses values of epidermis and dermis were noticeably thicker for mice treated with DNCB than those of normal skin. H&E stained sections showed dense infiltration of inflammatory cells into both epidermis and dermis regions of DNCB-treated mice (Figure 3(A)). In contrast, reduced levels of inflammatory cells were observed in FB801 and dexamethasone-treated atopic mice (Figure 3(A)). In addition, reduced epidermal thickening was observed in FB801 and dexamethasone-treated groups after oral administrations compared to that in DNCB-alone treated group of mice (Figure 3(B)). Similarly, increased levels of mast cell infiltration were observed in epidermis regions of DNCB-treated mice compared to the control group (Figures 3(A–C)). However, significantly decreased mast cell infiltration was observed in FB801-administered mice, similar to that in dexamethasone-treated mice (Figures 3(A–C)). These results strongly indicate that FB801 has *in vivo* pharmacological effects. It ameliorated AD symptoms at a dose of 30 mg/kg. Its effect was comparable to dexamethasone, a current anti-inflammatory drug for AD.

### Effects of FB801 on serum IgE and cytokines levels

**Figure 4.**
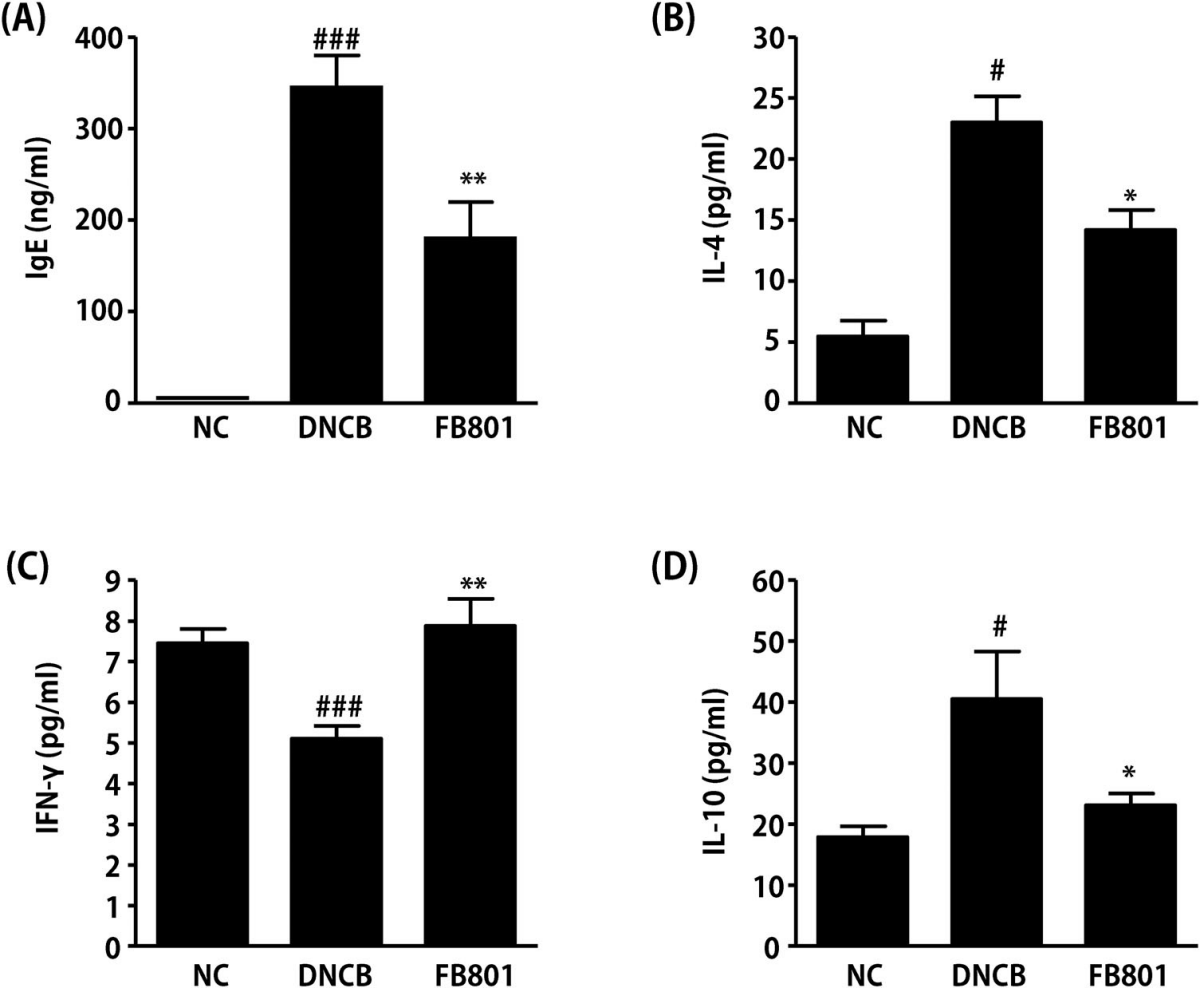
FB801 decreases serum levels of immunoglobulin E (IgE) and proinflammatory cytokines. Blood samples were collected from each mouse. Serum levels of (A) IgE, (B) interleukin (IL)-4, (C) interferon-γ, and (D) IL-10 were quantified by ELISA. Data are expressed as mean ± SEM of five mice per group. ^#^*P* < 0.05 and ^###^*P* < 0.001 compared to the normal control (NC) group. *P < 0.05 and **P < 0.01 compared to DNCB-stimulated group.

Increased total serum IgE level is a hallmark of AD. Thus, we next measured IgE levels in serum samples. Serum IgE levels in DNCB-treated mice were significantly increased than those in normal control mice (Figure 4(A)). However, DNCB-induced synthesis of IgE was significantly reduced by the administration of FB801. To evaluate the effects of FB801 treatments on Th1 and Th2 immunities, we measured levels of IL-4 and IFN-γ in sera samples of experimental mice. As shown in Figure 4(B), IL-4 levels were significantly increased in DNCB-treated mice, whereas IFN-γ levels were significantly decreased in DNCB-treated mice than in the normal group (Figure 4(C)). However, FB801 administration reversed these DNCB-induced changes of Th1 and Th2 cytokine levels in the serum. That is, FB801 treatment suppressed DNCB-induced increase of IL-4 levels (Figure 4(B)), whereas it increased IFN-γ levels in the DNCB group (Figure 4(C)). We also found that FB801 treatment reduced serum levels of IL-10 whose expression was induced after DNCB application (Figure 4(C)). These results suggest that FB801administration can suppress skin inflammation by inhibiting DNCB-stimulated increases of serum IgE and Th2-type cytokines.

### Effects of FB801 on TNF-α stimulated HaCaT cell proinflammatory cytokine expression

**Figure 5.**
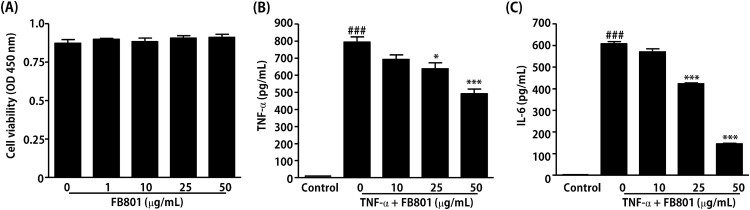
FB801 inhibits the inflammatory responses in tumor necrosis factor (TNF)-α-treated keratinocytes. (A) HaCaT cells were incubated with indicated concentrations of FB801 for 24 h, and cell viability was determined by Cell Counting Kit-8. HaCaT cells were pretreated with indicated concentrations of FB801 for 4 h and stimulated with TNF-α (10 ng/mL) for another 24 h. TNF-α (B) and interleukin-6 (C) levels were measured using culture supernatants by enzyme-linked immunosorbent assay. The values shown represent the mean ± SEM. *P < 0.05, ***P < 0.001, versus TNF-α-treated cells; ^###^*P* < 0.001 versus non-treated control cells.

The anti-AD efficacy of FB801 was evaluated using HaCaT cells to determine the detail molecular mechanism. First, the non-cytotoxic concentrations of FB801 to HaCaT cells were examined. As shown in Figure 5(A), concentrations of FB801 up to 50 µg/mL did not affect HaCaT cell viability. Therefore, we used up to 50 µg/mL of FB801 in further experiments.

Next, ELISA was performed to study the inhibitory effect of FB801 on the production of TNF-α and IL-6 in TNF-α-stimulated HaCaT cells. The results showed that TNF-α stimulation significantly increased the amounts of TNF-α and IL-6 in HaCaT cell culture medium compared with the untreated group, and these proinflammatory cytokines were significantly inhibited by treatment with 25 and 50 μg/mL of FB801 (Figure 5(B) and 5(C)).

### Effects of FB801 on the NF-kB and mitogen-activated protein kinase (MAPK) signaling pathway in TNF-α stimulated HaCaT cells

**Figure 6.**
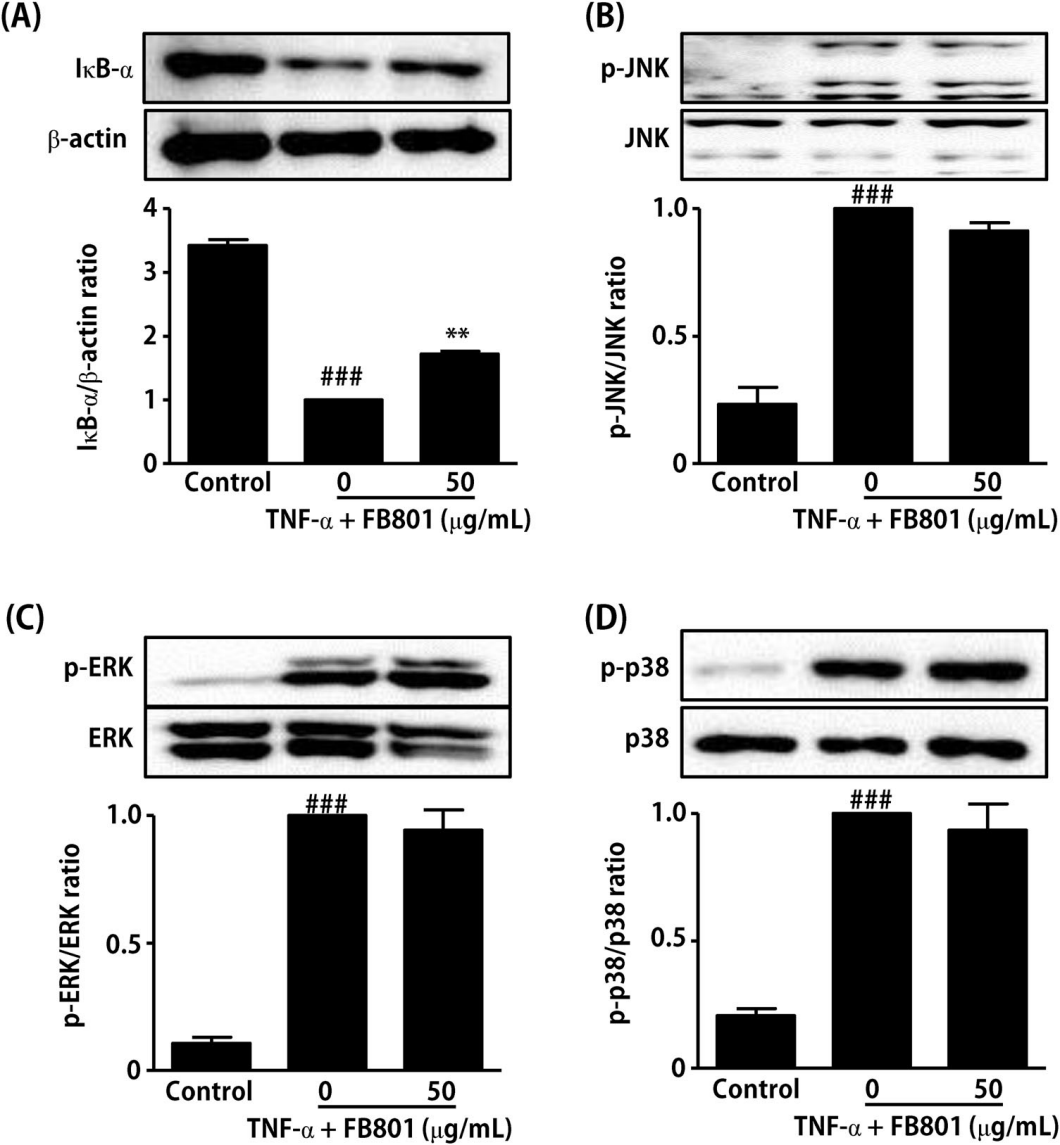
Effect of FB801 treatment on tumor necrosis factor (TNF)-α-induced nuclear factor-κB (NF-κB) and mitogen-activated protein kinase (MAPK) activation in HaCaT cells. HaCaT cells were treated with 50 μg/mL of FB801 for 4 h and stimulated with TNF-α (10 ng/mL) for another 15 min. (A) IκB-α protein levels were analyzed in whole cell lysates using Western blots, β-actin expression was used as an internal control. Phosphorylation and total protein expression of JNK (B), ERK 1/2 (C) and p38 (D) signaling proteins were analyzed in whole cell lysates using Western blot analysis. The values shown represent the mean ± SEM. **P < 0.001, versus TNF-α-treated cells; ^###^*P* < 0.001 versus non-treated control cells.

Since many previous studies have reported that activation of NF-κB and MAPK signaling pathways is involved in TNF-α-induced expression of proinflammatory mediators in keratinocytes (Razali et al. 2018; Sikandan et al. 2018; Hwang et al. 2020), we further examined the effect of FB801 on TNF-α-induced IκB-α degradation and phosphorylation of MAPK by Western blot analysis. Stimulation of HaCaT cells with TNF-α (10 ng/mL, 15 min) rapidly induced IκB-α degradation and MAPK phosphorylation, as shown in Figure 6. However, FB801 (50 µg/mL, 4 h) pretreatment significantly reduced IκB-α degradation in TNF-α-stimulated HaCaT cells (Figure 6(A)) but not phosphorylation of MAPK (Figure 6(B–C)). These results indicate that FB801 reduced the proinflammatory cytokines expressions through an alleviating effect on the NF-κB signaling pathway activated by TNF-α in HaCaT cells.

## Discussion

AD is an inflammatory skin disease provoked by an imbalance between Th1 and Th2 immune responses. It is one of the most common skin lesions in pediatric patients with a genetic predisposition (Custovic et al. 2015; Weidinger and Novak 2016; Furue et al. 2017; Kim et al. 2019). AD can prompt edema, erythema, itching, skin pigmentation, thickening, eczematous lesions, and excoriation of the skin. These symptoms might be accompanied by sleep disturbance and depression. They can also affect the quality of life of patients (Koszorú et al. 2019). AD is also frequently associated with food allergy, which complicates its management in approximately 40% of children (Bergmann et al. 2013). It is related to hyperresponsiveness of lymphocytes to allergens. Furthermore, mast cell degranulation and production of histamine and IgE are closely associated with allergic reactions (Custovic et al. 2015; Weidinger and Novak 2016; Furue et al. 2017; Kim et al. 2019). Currently, steroids, antihistamines, and immunosuppressive agents are mainly used to treat AD, but with side effects (Megna et al. 2017).

Functional properties of purple corn have been progressively studied. More than 20 bioactive phenolic compounds, including phenolic acids, anthocyanins, and other flavonoids, have been reported to be found in purple corn (Francavilla and Joye 2020). Numerous studies have shown that these phenolic compounds of purple corn possess potent health-benefitting capabilities, including antioxidant, anti-inflammatory, anti-mutagenic, anti-carcinogenic, anti-cancer, and anti-angiogenesis properties that can ameliorate lifestyle diseases such as obesity, diabetes, hyperglycemia, hypertension, and cardiovascular diseases (Marunaka et al. 2017; Costea et al. 2018; Yahfoufi et al. 2018). These beneficial properties of purple corn are largely attributed to strong antioxidant capabilities and anti-inflammatory properties of its phenolic compounds. Based on this, we investigated the anti-atopic effect of FB801, a novel purple corn extract, and confirmed the possibility of using it as a therapeutic agent in the treatment of AD.

Various reports have demonstrated that Th2-type immune responses play significant roles in the pathogenesis of AD (Weidinger and Novak 2016; Kim et al. 2019). Th2 cytokines are considered to be attractive therapeutic targets for AD treatment. In AD skin lesions, Th2 lymphocytes produce high levels of IL-4, IL-5, IL-6, IL-10, and IL-13, which can induce IgE production by B cells. IgE in turn can bind to high-affinity IgE-Fc receptor type I on the surface of mast cells, leading to the release of various types of inflammatory mediators such as histamine, chemokines, and cytokines (Custovic et al. 2015; Weidinger and Novak 2016; Kim et al. 2019; Furue 2020). IL-4 is also a major driving factor for Th2 responses known to cause AD (Chiricozzi et al. 2020). When its expression and secretion are raised in immune cells, the response of sub-signals such as signal transducer and activator of transcription 6 phosphorylation will increase and symptoms of AD will appear. The efficacy of dupilumab, a drug that modulates IL-4, is known to be the best in a clinical trial (Vangipuram and Tyring 2017; Rodrigues et al. 2019). Previous works have demonstrated that extracts of natural products can inhibit IL-4 expression and that some compounds from these extracts have excellent efficacies against AD in animal models (Jung et al. 2016; Furue 2020). Therefore, it can be inferred that compounds that can inhibit IL-4 and IgE expression can ameliorate AD in humans.

Keratinocytes such as HaCaT cells play central roles in skin disease, the initiation and maintenance of AD (Chieosilapatham et al. 2021). Therefore, HaCaT cells are commonly used for the *in vitro* testing of anti-inflammatory and anti-atopic skin agents (Colombo et al. 2017). It has been reported that stimulation of keratinocytes by TNF-α increases the expression of proinflammatory mediators through NF-κB and MAPK signaling pathways (Razali et al. 2018; Sikandan et al. 2018; Hwang et al. 2020). Based on these facts, we examined the direct effects of FB801 on human keratinocytes. Treatment of TNF-α significantly enhanced the phosphorylation of MAPKs and degradation of IκB-α whereas FB801 treatment significantly inhibited IκB-α degradation but not the phosphorylation of MAPK in HaCaT cells stimulated with TNF-α. These results suggest that the inhibitory effect of FB801 on skin inflammation can be achieved, at least in part, through inhibition of the NF-κB signaling pathway.

It is important to accurately identify which components of FB801 exhibit anti-AD activity. Previous studies including ours, have shown that *Zea mays* L. contains a variety of potentially active compounds that modulate AD symptoms (Kim et al. 2013; Lao et al. 2017). FB801 contains at least 15 compounds that may exhibit anti-AD activity (Supplementary material). Among them, cyanidin-3-glucoside, the most abundant anthocyanin in FB801, was found to activate Th2 by down-regulating Th2 cytokines and GATA3 transcription factors in activated EL-4 T cells (Pyo et al. 2014). In addition, administration of ferulic acid ameliorated the overall symptoms of AD by inhibiting Th2 cytokines and IgE in DNCB-treated mice (Zhou et al. 2020). It is currently unclear whether a certain compound contained in FB801 exhibits anti-AD activity or several compounds interact to exhibit anti-AD activity. However, research is currently underway to find the anti-AD component contained in FB801 and will be reported in the near future.

## Conclusion

Our findings showed that oral administration of FB801 attenuated DNCB-induced AD-like symptoms in BALB/c mice. FB801 treatment significantly alleviated DNCB-induced increase in skin lesion severity, dermatitis score, ear thickness, and scratching behavior. In addition, DNCB-induced infiltration of skin lesions by mast cells was decreased following oral treatment of FB801. We also found that FB801 reduced serum levels of IgE and Th2 cytokines such as IL-4 and IL-10, with a concomitant increase of IFN-γ in DNCB-treated BALB/c mice. Although further studies are needed to investigate the clinical usefulness of FB801 against AD symptoms in human, these findings suggest that oral administration of FB801 might be useful for the treatment of AD.

## Supplementary Material

Supplemental Material
