# Supplementary material for: Purple corn extract alleviates 2,4-dinitrochlorobenzene-induced atopic dermatitis-like phenotypes in BALB/c mice
Source: Anim Cells Syst (Seoul). 2021 Sep 8;25(5):272–82. doi: 10.1080/19768354.2021.1974938 (PMC8567911; doi:10.1080/19768354.2021.1974938)
Supplement: Supplemental Material [file TACS_A_1974938_SM0913.docx]

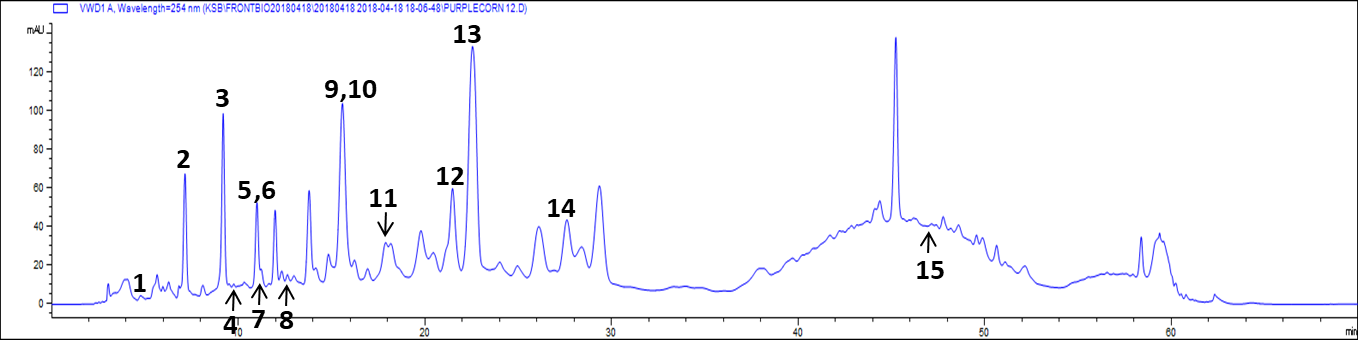


**Figure 1. HPLC chromatography of FB801. Fifteen compounds constituting FB801 shown in Table 1.**

**Table. 1. The anti-atopic and anti-inflammatory effects of 15 major compounds constituting FB801 and their effects on IgE production were summarized through literature search.**

| **NO** | **Name** | **Anti-atopic effect** | **Anti-inflammatory**  **effect** | **TH_1_**  **cytokines** | **TH_2_ cytokines** | **IgE** |
| --- | --- | --- | --- | --- | --- | --- |
| **1** | **Gallic acid ^1-5)^** | ○^a^ | ○ | ↓ ^c^ | ↓ | - |
| **2** | **Protocatechuic acid ^6-10, 26)^** | -^b^ | ○ | ↓ | ↓ | ↓ |
| **3** | **4-hydroxybenzoic acid** | - | - | - | - | - |
| **4** | **p-hydroxyphenyl acetic acid** | - | - | - | - | - |
| **5** | **Vanillic acid ^11-14)^** | - | ○ | ↑^d^ | ↓ | - |
| **6** | **Chlorogenic acid ^1, 14-17)^** | ○ | ○ | ↓ | ↓ | ↓ |
| **7** | **Caffeic acid ^14, 18-21)^** | ○ | ○ | - | ↓ | - |
| **8** | **Syringic acid ^22-23)^** | - | ○ | ↓ | ↓ | - |
| **9** | **Cyanidin-3-glucoside ^24-27)^** | - | ○ | - | ↓ | - |
| **10** | ***p*-coumaric acid ^14, 28-29)^** | ○ | ○ | ↑ | ↓ | ↑ |
| **11** | **Trans-ferulic acid ^14, 30-35)^** | ○ | ○ | ↓ | ↓ | - |
| **12** | **Rutin ^36-40)^** | ○ | ○ | ↓ | ↓ | ↓ |
| **13** | **Isoquercitrin ^41-44)^** | - | ○ | ↑ | ↓ | ↓ |
| **14** | **Quercetin ^42, 45-50)^** | ○ | ○ | ↓ | ↓ | ↓ |
| **15** | **Kaempferol ^45, 51-59)^** | ○ | ○ | ↑ | ↓ | ↓ |

^a^○ indicates that there are papers related to anti-atopic and anti-inflammatory effects. ^b^- indicates that there is no data. ^c^↓ indicates downregulated and ^d^↑ indicates upregulated. Reference is indicated by footnote number after each compound name.

**[HPLC method]**

HPLC equipment was an Agilent 1200 series instrument (Agilent Technologies, Seoul, Korea) consisting of a vacuum degasser (G1322A), a quaternary pump (G1311A), an auto-sampler (G1329A), a thermostatted column compartment (TCC, G1316A), and a variable wavelength detector (VWD, G1314D) system. HPLC were achieved using a Agilent Edipse XDB-Phenyl 4.6X150 mm (3.5um). The mobile phase, consisting of 0.1% aqueous trifluoroacetic acid and acetonitrile, was used at a flow rate of 0.7 mL min-1. The gradient elution program was modified as follows for a total of 70min : 0–10% B (0–5 min), 10–15% B (5–20 min), 15–15% B (20–32 min), 15–30% B (32–45 min), 30–30% B (45–50 min), 30–40% B (50–55 min), 40–100% B (55–58 min), 100–0% B (58–60 min), and 0–0% B (60–70 min). Injection volume was 10 μL at a sample concentration 1 mg/mL in methanol, and the detection wavelength was 254 nm.

**[Reference]**

1. Miranda S. M. Tsang et al. 2016. Anti-Inflammatory Activities of Pentaherbs Formula, Berberine, Gallic Acid and Chlorogenic Acid in Atopic Dermatitis-Like Skin Inflammation. molecules. 21(4):519.
2. Chong-Hyeon Yoon et al. 2013. Gallic acid, a natural polyphenolic acid, induces apoptosis and inhibits proinflammatory gene expressions in rheumatoid arthritis fibroblast-like synoviocytes. Joint Bone Spine. 80:274-279.
3. Herbert Kolodziej et al. 2008. Stimulus (polyphenol, IFN-c, LPS)-dependent nitric oxide production and antileishmanial effects in RAW 264.7 macrophages. Phytochemistry. 69:3103-3110.
4. Kei KATO et al. 2001. Effect of Gallic Acid Derivatives on Secretion of Th1 Cytokines and Th2 Cytokines from Anti CD3-Stimulated Spleen Cells. YAKUGAKU ZASSHI. 121(6):451-457.
5. Seok-Bin Yoon et al. 2009. Anti-inflammatory effects of Scutellaria baicalensis water extract on LPS-activated RAW264.7 macrophages. Journal of Ethnopharmacology. 125:286-290.
6. YOSHIMASA NAKAMURA et al. 2001. A CATECHOL ANTIOXIDANT PROTOCATECHUIC ACID POTENTIATES INFLAMMATORY LEUKOCYTE-DERIVED OXIDATIVE STRESS IN MOUSE SKIN VIA A TYROSINASE BIOACTIVATION PATHWAYs. Free Radical Biology & Medicine. 30(9):967-978.
7. Miaomiao Wei et al. 2013. Protocatechuic acid suppresses ovalbumin-induced airway inflammation in a mouse allergic asthma model. International Immunopharmacology. 15:780-788.
8. Sahil Kakkar and Souravh Bais. 2014. A Review on Protocatechuic Acid and Its Pharmacological Potential. ISRN Pharmacology. 9.
9. Changbo Ou et al. 2014. Protocatechuic Acid, a Novel Active Substance against Avian Influenza Virus H9N2 Infection. PLoS. 9(10).
10. S-J Han et al. 2009. Metabolism of Cyanidin-3-O-β-D-Glucoside Isolated from Black Colored Rice and Its Antiscratching Behavioral Effect in Mice. JOURNAL OF FOOD SCIENCE. 74(8):253-258.
11. Ponnian Stanely Mainzen Prince et al. 2011. Protective effects of vanillic acid on electrocardiogram, lipid peroxidation, antioxidants, proinflammatory markers and histopathology in isoproterenol induced cardiotoxic rats. European Journal of Pharmacology. 668:233-240.
12. Min-Cheol Kim et al. 2011. Vanillic acid inhibits inflammatory mediators by suppressing NF-κB in lipopolysaccharide-stimulated mouse peritoneal macrophages. Immunopharmacology and Immunotoxicology. 33(3):525–532.
13. Ganapathy Sindhu et al. 2015. Nephroprotective effect of vanillic acid against cisplatin induced nephrotoxicity in wistar rats: a biochemical and molecular study. Environ Toxicol Pharmacol. 39(1):392-404.
14. Lien-Chai Chiang et al. 2003. Immunomodulatory activities of flavonoids, monoterpenoids, triterpenoids, iridoid glycosides and phenolic compounds of Plantago species. Planta Med. 69(7):600-604.
15. Su Jung Hwang et al. 2014. Anti-inflammatory effects of chlorogenic acid in lipopolysaccharide-stimulated RAW 264.7 cells. Inflamm. Res. 63:81–90.
16. Xiang Li et al. 2013. Chlorogenic Acid Inhibits the Replication and Viability of Enterovirus 71 In Vitro. PLoS One. 30;8(9):e76007.
17. Hye-Rin Kim et al. 2010. Chlorogenic acid suppresses pulmonary eosinophilia, IgE production, and Th2-type cytokine production in an ovalbumin-induced allergic asthma: Activation of STAT-6 and JNK is inhibited by chlorogenic acid. International Immunopharmacology. 10:1242–1248.
18. Maria Alejandra HOSSEN et al. 2006. Caffeic Acid Inhibits Compound 48/80-Induced Allergic Symptoms in Mice. Biol Pharm Bull. 29(1):64-66.
19. Mengjun Zhang et al. 2014. Caffeic Acid Reduces Cutaneous Tumor Necrosis Factor Alpha (TNF-α), IL-6 and IL-1β Levels and Ameliorates Skin Edema in Acute and Chronic Model of Cutaneous Inflammation in Mice. Biol Pharm Bull. 37(3):347–354.
20. Shih-jei Tsai et al. 2011. Preventive and therapeutic effects of caffeic acid against inflammatory injury in striatum of MPTP-treated mice. European Journal of Pharmacology 670:441–447.
21. Yong-Deok Jeon et al. 2015. Effects of Ixeris dentata water extract and caffeic acid on allergic inflammation in vivo and in vitro. BMC Complementary and Alternative Medicine. 15:196.
22. Ju Ri Ham et al. 2016. Anti-steatotic and anti-inflammatory roles of syringic acid in high-fat diet-induced obese mice. Food Funct. 7(2):689-697.
23. Ayano ITOH et al. 2009. Hepatoprotective Effect of Syringic Acid and Vanillic Acid on Concanavalin A-Induced Liver Injury. Biol Pharm Bull. 32(7):1215-1219.
24. Yunhe Fu et al. 2014. Cyanidin-3-O-β-glucoside inhibits lipopolysaccharide-induced inflammatory response in mouse mastitis model. J Lipid Res. 55(6):1111-1119.
25. Yinghui Zhang et al. 2010. Cyanidin-3-O-b-glucoside inhibits LPS-induced expression of inflammatory mediators through decreasing IjBa phosphorylation in THP-1 cells. Inflamm. Res. 59:723–730.
26. Sung-Won Min et al. 2010. Anti-inflammatory effects of black rice, cyanidin-3-O-β-D-glycoside, and its metabolites, cyanidin and protocatechuic acid. International Immunopharmacology. 10:959–966.
27. Hana Jung et al. 2014. Antioxidant and Antiinflammatory Activities of Cyanidin-3-glucoside and Cyanidin-3-rutinoside in Hydrogen Peroxide and Lipopolysaccharide-treated RAW264.7 Cells. Food Sci. Biotechnol. 23(6):2053-2062.
28. Phil-Dong Moon et al. 2021. p-coumaric acid, an active ingredient of Panax ginseng, ameliolates atopic dermatitis-like skin lesions through inhibition of thymic stromal lymphopoietin in mice. J Ginseng Res. 45(1):176-182.
29. Hua Li et al. 2014. Antioxidant and Anti-inflammatory Activities of Methanol Extracts of Tremella fuciformis and Its Major Phenolic Acids. J Food Sci. 79(4):C460-468.
30. Zhike Zhou et al. 2020. Ferulic acid alleviates atopic dermatitis-like symptoms in mice via its potent anti-inflammatory effect. Immunopharmacol Immunotoxicol. 42(2):156-164.
31. Nadia Lampiasi and Giovanna Montana. 2016. The molecular events behind ferulic acid mediated modulation of Il-6 expression in LPS-activated raw 264 cells. Immunobiology. 221(3):486-493.
32. Chengwei Niu et al. 2016. Ferulic acid prevents liver injury induced by Diosbulbin B and its mechanism. BioScience Trends. 10(5):386-391.
33. Simón Navarrete et al. 2015. Aqueous Extract of Tomato (Solanum lycopersicum L.) and Ferulic Acid Reduce the Expression of TNF-α and IL-1β in LPS-Activated Macrophages. Molecules. 20:15319-15329.
34. Yan-jun Cao et al. 2015. Ferulic acid inhibits H2O2-induced oxidative stress and inflammation in rat vascular smooth muscle cells via inhibition of the NADPH oxidase and NF-κB pathway. Int Immunopharmacol. 28(2):1018-1025.
35. Alessa Sin Singer Brugiolo et al. 2017. Ferulic acid supresses Th2 immune response and prevents remodeling in ovalbumin-induced pulmonary allergy associated with inhibition of epithelial-derived cytokines. Pulmonary Pharmacology & Therapeutics. 45:202-209.
36. Jin Kyeong Choi and Sang-Hyun Kim. 2013. Rutin suppresses atopic dermatitis and allergic contact dermatitis. Exp Biol Med (Maywood). 238(4):410-417
37. Peng-xin Xu et al. 2014. Rutin improves spatial memory in Alzheimer’s disease transgenic mice by reducing Aoligomer level and attenuating oxidative stress and neuroinflammation. Behavioural Brain Research. 264:173–180.
38. Wonhwa Lee et al. 2012. Barrier protective effects of rutin in LPS-induced inflammation in vitro and in vivo. Food and Chemical Toxicology. 50:3048–3055.
39. Eun Sun Yu et al. 2008. Regulatory mechanisms of IL-2 and IFNg suppression by quercetin in T helper cells. Biochemical pharmacology. 76:70-78.
40. Netaji T Niture et al. 2014. Anti-hyperglycemic activity of rutin in streptozotocin-induced diabetic rats: an effect mediated through cytokines, antioxidants and lipid biomarkers. Indian J Exp Biol. 52(7):720-727.
41. Kyung-A Hwang et al. 2018. Anti-allergic effect of Aster yomena on ovalbumin-sensitized mouse and RHL-2H3 cells via Th1/Th2 cytokine balance. Journal of Functional Foods. 44:1-8.
42. A. P. Rogerio et al. 2007. Anti-infl ammatory activity of quercetin and isoquercitrin in experimental murine allergic asthma. Infl amm res. 56:402-408.
43. Wenyan Xie et al. 2016. Hepatoprotective effect of isoquercitrin against acetaminophen-induced liver injury. Life Sci. 1(152):180-189.
44. A Ryun Kim et al. 2013. Phenolic compounds with IL-6 inhibitory activity from Aster yomena. Arch Pharm Res. 37(7):845-851.
45. Eun-Ju Lee et al. 2010. Quercetin and kaempferol suppress immunoglobulin E-mediated allergic inflammation in RBL-2H3 and Caco-2 cells. Inflamm Res. 59:847-854.
46. Vengadeshprabhu Karuppagounder et al. 2015. Modulation of HMGB1 translocation and RAGE/NFκB cascade by quercetin treatment mitigates atopic dermatitis in NC/Nga transgenic mice. Exp Dermatol. 24(6):418-423.
47. Jiajia Liu et al. 2005. The inhibitory effect of quercetin on IL-6 production by LPS-stimulated neutrophils. Cell Mol Immunol. 2(6):455-460.
48. Monica Comalada et al. 2005. In vivo quercitrin anti-inflammatory effect involves release of quercetin, which inhibits inflammation through down-regulation of the NF-jB pathway. Eur J Immunol. 35:584–592.
49. Xi Chen. 2010. Protective effects of quercetin on liver injury induced by ethanol. Pharmacogn Mag. 6(22):135-141.
50. Tatiane Teixeira Oliveira et al. 2015. Potential therapeutic effect of Allium cepa L. and quercetin in a murine model of Blomia tropicalis induced asthma. DARU Journal of Pharmaceutical Sciences. 23:18.
51. Hyun-Su Lee and Gil-Saeng Jeong. 2021. Therapeutic effect of kaempferol on atopic dermatitis by attenuation of T cell activity via interaction with multidrug resistance-associated protein 1. Br J Pharmacol. 178(8):1772-1788.
52. Xiaojun Chen et al. 2012. Kaempferol regulates MAPKs and NF-κB signaling pathways to attenuate LPS-induced acute lung injury in mice. International Immunopharmacology. 14:209-216.
53. Hyun-A Oh et al. 2013. Evaluation of the effect of kaempferol in a murine allergic rhinitis model. European Journal of Pharmacology. 718:48-56.
54. Qi-Sheng Yang et al. 2015. Kaempferol pretreatment modulates systemic inflammation and oxidative stress following hemorrhagic shock in mice. Chinese Medicine. 10:6.
55. Jose R Cortes et al. 2007. Kaempferol Inhibits IL-4-Induced STAT6 Activation by Specifically Targeting JAK3^1^. J Immunol. 179(6):3881-3887.
56. Mi Ja Chung et al. 2015. Inhibitory effects of kaempferol-3-O-rhamnoside on ovalbumin-induced lung inflammation in a mouse model of allergic asthma. International Immunopharmacology. 25:302–310.
57. Myungsuk Kim et al. 2016. Inhibitory effect of the Larix sibirica and its various flavonoids on the IgE-stimulated mast cell activation and anaphylaxis. Journal of Functional Foods. 27:631–644.
58. K Y Park et al. 1999. Inhibitory effect of luteolin 4'-O-glucoside from Kummerowia striata and other flavonoids on interleukin-5 bioactivity. Planta Med. 65(5):457-459.
59. Marta Palacz-Wrobel et al. 2017. Effect of apigenin, kaempferol and resveratrol on the gene expression and protein secretion of tumor necrosis factor alpha (TNF-a) and interleukin-10 (IL-10) in RAW-264.7 macrophages. Biomedicine & Pharmacotherapy. 93:1205-1212.
